# Pattern of Cytopathic Effects Produced by Recent Bangladeshi Field Isolates of PPR Virus in Vero Cells and Preparation of Purified Viral Antigen

**DOI:** 10.1155/vmi/9442403

**Published:** 2026-09-18

**Authors:** M. S. I. Siddiqui, Jahan Ara Begum, M. R. Islam, E. H. Chowdhury

**Affiliations:** ^1^ Department of Anatomy & Histology, Faculty of Veterinary, Animal & Biomedical Sciences, Sylhet Agricultural University, Sylhet 3100, Bangladesh, sau.ac.bd; ^2^ Department of Pathology, Faculty of Veterinary Sciences, Bangladesh Agricultural University, Mymensingh 2202, Bangladesh, bau.edu.bd

**Keywords:** CPE dynamics, LKC, PPR virus, purified viral antigen, quantitation, Vero cells

## Abstract

**Background:**

This study was conducted to explore the pattern of appearance of cytopathic effect (CPE) produced by a Bangladeshi strain of Peste des petits ruminant’s virus (PPRV) in Vero cells during serial passaging, to estimate the virus titer and concentration of PPR viral RNA at different passage levels, and to find out a suitable passage level for viral antigen purification.

**Methods:**

Five isolates of PPR virus from local outbreaks were isolated, confirmed with conventional RT‐PCR, and then used as viral inoculant in Vero cells and lamb kidney cell culture (LKC). Morphology of CPE’s and pattern of development of CPE were studied and recorded at different passage levels and days postinfection (dpi). Tissue culture fluid (TCF) was used to prepare purified viral antigen by ultracentrifugation and for quantitation by spectrophotometry. Viral titers were determined following an endpoint dilution assay.

**Results:**

The study revealed that the CPE was not perceptible in all isolates until 11 dpi for the first six passages. The virus was not detected in TCF of both infected and control flasks by RT‐PCR up to the 6th passage. The first CPE appeared on 5 dpi at the 7th passage level for all isolates. The CPE was characterized by initial cell rounding, followed by the formation of small syncytia, progression to total cell infection, and eventual complete detachment of the cell monolayer. Maximum titer (3.5 Log_10_TCID_50_) and RNA concentration (260 ng/µL) were found at the 30^th^ passage level by one isolate (Isolate‐F).

**Conclusion:**

It is concluded that initiation of CPE production in Vero cells by the Bangladeshi field strain of PPR virus occurs at the 7th passage level. Virus titer and concentration of PPR viral RNA increased with the advancement of passaging, and these are highly related to each other. TCF at higher passage levels, such as the 60^th^ passage, is most suitable for purification of PPR viral antigen.

**Summary**
• Serial passaging of field isolates of Bangladeshi strain PPR virus did not produce cytopathic effect (CPE) in Vero cells up to 11 days postinfection (dpi) for the first six passages.
• The virus was not detected in the fluid of infected flasks (TCF) by conventional RT‐PCR up to the 6^th^ passage.
• The first CPE appeared on 5 dpi at the 7^th^ passage level.
• The infected flasks were harvested at 11 dpi, and virus was detected in the fluid of infected flasks at the 7^th^ passage by conventional RT‐PCR.
• Titers produced by different field isolates of Bangladeshi PPR virus on specific passage levels are very close to each other. The maximum titer (3.5 Log_10_TCID_50_) and RNA concentration (260 ng/µL) were found at the 30^th^ passage level by one isolate (Isolate‐F).
• At the 60^th^ passage level, the concentration of viral RNA is much higher, so the TCF of this passage level can be used for the preparation of purified PPR viral antigen

## 1. Introduction

Peste des petits ruminants (PPR) is a highly contagious viral disease of sheep and goats that causes substantial economic losses through high morbidity, mortality, and reduced livestock productivity. Because of its serious socioeconomic impact and transboundary nature, PPR has been recognized as a transboundary animal disease by the World Organization for Animal Health [1]. The causative agent, PPR virus (PPRV), belongs to the genus Morbillivirus within the family Paramyxoviridae. Based on genetic characterization, PPRV is classified into four distinct lineages (I–IV), of which lineage IV has become the predominant lineage throughout Asia, including Bangladesh [2]. Continuous viral evolution through mutation and adaptation results in genetic variation among circulating field isolates, which may influence viral pathogenicity, host adaptation, and epidemiological characteristics. Consequently, characterization of currently circulating field isolates is essential for understanding virus biology and for developing effective vaccines and diagnostic tools [3].

Isolation and propagation of field isolates in cell culture constitute an important step in virological investigations because they provide sufficient viral material for vaccine development, molecular characterization, antigen production, and immunological studies. Among the available cell culture systems, Vero cells are widely used for the isolation, propagation, and identification of PPRV owing to their high susceptibility after viral adaptation [4–7]. During replication in cultured cells, PPR virus induces characteristic cytopathic effects (CPE), including cell rounding, syncytium formation, intranuclear and intracytoplasmic inclusion bodies, cellular degeneration, and eventual detachment of the cell monolayer. These morphological changes, typically observed using a phase‐contrast inverted microscope, provide valuable indicators of successful viral replication and adaptation in vitro [8–11].

Although Vero cells are extensively employed for PPRV propagation, they are not the natural host cells of the virus [12]. Consequently, adaptation of field isolates to Vero cells is often slow and requires multiple serial passages before consistent viral replication and visible CPE can be observed [13]. The onset of CPE varies considerably among virus isolates and may depend on viral lineage, strain characteristics, and geographical origin [7, 12]. Parameters such as the passage number at which CPE first appears, the hours postinoculation (hpi) required for initial cellular changes, the dpi at which CPE becomes detectable, and the optimal harvesting time of tissue culture fluid (TCF) may, therefore, differ substantially among isolates [13, 14]. Furthermore, during the early stages of serial passaging, viral RNA may remain undetectable in TCF by conventional or real‐time RT‐PCR despite the presence of infectious virus, making it difficult to determine whether successful adaptation has occurred [4, 7, 15]. These uncertainties often complicate virus isolation and delay subsequent virological studies [7].

As serial passaging progresses, viral adaptation generally leads to increased viral replication and higher virus titers. Consequently, both viral particle concentration and viral RNA abundance in TCF increase with advancing passage levels. Quantification of viral nucleic acid is, therefore, an important indicator of viral replication and adaptation and provides valuable information for genomic studies and disease diagnosis [16, 17]. In addition, high‐quality purified viral preparations are required for complete genome sequencing, protein characterization, immunological investigations, and the development of antigen‐based diagnostic assays [18]. Successive ultracentrifugation remains an effective method for concentrating and purifying virus particles to obtain high‐titer viral preparations suitable for these applications [19].

Despite the importance of virus adaptation in Vero cells, limited information is available regarding the adaptation characteristics of recent Bangladeshi field isolates of PPRV. In particular, no comprehensive study has determined the earliest passage at which CPE appears, the specific hpi and dpi associated with the initiation of CPE, or the relationship between serial passage and viral RNA concentration in TCF. Such information is essential for optimizing virus propagation protocols, producing purified viral antigen, and improving the development of diagnostic reagents and future vaccine candidates.

Therefore, the present study was undertaken to determine the earliest passage at which CPE becomes detectable in Vero cells and viral RNA can be identified in TCF from recent Bangladeshi field isolates of PPRV. The study also aimed to characterize the timing of CPE development in terms of hpi and dpi, quantify viral RNA at different serial passage levels, and prepare purified PPR viral antigen for downstream diagnostic and research applications.

## 2. Materials and Methods

### 2.1. Materials

#### 2.1.1. Virus Samples

Bronchial and mesenteric lymph nodes were taken as samples after postmortem examination of PPR‐suspected dead goats. RT‐PCR was performed for initial confirmation of the virus. Only the samples that tested positive were selected for the study. The isolates were designated as A–F for ease of reference as follows: BD‐PPR‐BAU‐GF‐01‐13 (A), BD‐PPR‐BAU‐CL‐01‐13 (B), BD‐PPR‐BAU‐CL‐03‐13 (C), BD‐PPR‐BAU‐CL‐06‐13 (D), BD‐PPR‐BAU‐CL‐07‐13 (E), and BD‐PPR‐08 (F), the latter obtained from a virus repository from a previous study at the 9^th^ passage level.

#### 2.1.2. Vero Cells

For isolation and identification of PPR virus from field samples, Vero cells obtained as a subculture from Cell Line Services (CLS), Germany, were used for this study.

#### 2.1.3. Establishment of Lamb Kidney Primary Cell Culture for Isolation of PPR Virus

Lamb kidney primary cell culture (LKC) was established following the protocol of [20] and used for isolation of PPR virus and comparative study of the CPE produced by PPR virus in Vero cells.

#### 2.1.4. Cell Culture Media and Reagents

Cell culture medium‐M‐199 (Gibco‐Invitrogen, cat no. 11825) and calf serum–FBS (Gibco‐Invitrogen, cat no. 10437) were used for culture and maintenance of Vero cells. Cell culture working medium was prepared by adding L‐glutamine, HEPES buffer, sodium bicarbonate, antifungal solution (Fungizone), antibiotic (gentamicin), and distilled water with commercially available M‐199 solution. 0.25% Trypsin with EDTA (Gibco‐Life Technologies, 20,367, C13) was used in this study for dissociation and detachment of cells from the cell culture flask during subculture.

### 2.2. Methods

#### 2.2.1. Sources of Different Bangladeshi Field Isolates

Isolates were collected from different PPR outbreaks that occurred in different areas adjacent to the study area or institutes. The history of all outbreaks was recorded and is shown in Table 1.

**TABLE 1:** History of field PPR virus isolates used in the serial passaging of this study.

SL	Isolates ID	Location of PPR outbreaks	Date of sample collection	Animals—goat (*Capra hircus*)	Clinical signs	Other information’s	Types of tissue collected
01	Isolate‐A	Bangladesh Agricultural University (BAU) goat farm	19.01.2013	Breed: Black Bengal goat Age: 1 year	Fever (100.5°F), cough, conjunctivitis, dyspnea, stomatitis, diarrhea, and death	Animals in the herd: 12, animals affected: 12, died: 7 No vaccination	Lymph node^∗∗^ (bronchial, mesenteric, prescapular lymph node), trachea, lungs, and intestine

02	Isolate‐B	Digarkanda, Thana: Kotowali, District: Mymensingh (Sick goat attended at BAU Veterinary Teaching Hospital and died)	13.01.2013	Breed: Black Bengal goat Age: 1.5 years	Fever (100°F), mucopurulent oculo‐nasal discharge, dyspnea, stomatitis, severe diarrhea, dehydration, and death	Animals in the herd: 3, Animals affected: 3, No vaccination	Do

03	Isolate‐C	Boira, Thana: Kotowali, District: Mymensingh (Sick goat attended at BAU Veterinary Teaching Hospital and died)	29.01.2013	Breed: Black Bengal goat Age: 1.2 years	Fever (101°F), mucopurulent oculo‐nasal discharge, dyspnea, stomatitis, severe diarrhea, dehydration, and death	Animals in the herd: 7, Animals affected: 5, died: 2 No vaccination	Do

04	Isolate‐D	Kawatkhali, Thana: Kotowali, District: Mymensingh (Sick goat attended at BAU Veterinary Teaching Hospital and died)	11.02.2013	Breed: Black Bengal goat Age: 1.6 years	Fever (100°F), mucopurulent oculo‐nasal discharge, dyspnea, stomatitis, severe diarrhea, dehydration, and death	Animals in the herd: 2, Animals affected: 1, died: 1, No vaccination	Do

05	Isolate‐E	Boira, Thana: Kotowali, District: Mymensingh (Sick goat attended at BAU Veterinary Teaching Hospital and died)	13.02.2013	Breed: Black Bengal goat Age: 1.6 years	Fever (100°F), mucopurulent oculo‐nasal discharge, dyspnea, stomatitis, severe diarrhea, dehydration, and death	Animals in the herd: 2, Animals affected: 1, died: 1, No vaccination	Do

06	Isolate‐F^∗^	Karwan Bazar, Dhaka	23.03.2008	Breed: Black Bengal goat Age: 1.5 years	Fever (100°F), sneezing, depression, anorexia, oculo‐nasal discharge, dyspnea, stomatitis, diarrhea, and death	Animals in the herd: 16, animals affected: 9, died: 7, No vaccination	Do

^∗^Finally selected as vaccine candidate (Collected from virus repository).

^∗∗^Bronchial lymph node suspension was used for initial virus isolation.

#### 2.2.2. Isolation of Bangladeshi Field Isolates of PPR Virus

##### 2.2.2.1. Preparedness of Viral Inoculum

Collected mesenteric and bronchial lymph nodes were smashed with a sterile mortar and pestle; PBS was added, centrifuged, and then an antibiotic (Gentamicin) was added. Tissue suspension was filtered using an Acrodisc Syringe Filter (0.2 μm size, Cytiva, Sweden), and the filtrate was used as viral inoculum.

##### 2.2.2.2. Inoculation of Virus in Vero Cells and Lamb Kidney Cells (LKC)

The confluent monolayer of Vero cells (90^th^ subculture) and LKC (5^th^ subculture) was washed with PBS; the medium was discarded and then inoculated with viral inoculum at a fixed multiplicity of infection (MOI) of 0.1 at 200 μL/25 sq. cm cell culture flasks. An equal amount of PBS was used in the control flask as a mock infection. Both infected and control flasks were placed at 37°C in an incubator and spread by tilting for 1 h for adsorption of the virus. Five‐percent newborn calf serum (Gibco, USA) was added as maintenance medium, and the flasks were incubated again and examined twice daily for CPE study with the help of a phase‐contrast inverted microscope (Olympus, Japan).

#### 2.2.3. Study on Dynamics or Pattern of Production of CPE in Vero Cells

The dynamics or pattern of production of CPE of PPR virus on Vero cells such as first time appearance of CPE (both passage level and hpi or dpi, percentage of dead or floating cells, percentage of rounded cells, time of appearance of small syncytia, time of large syncytia development, time of approximately 100% cell rounding, and time of complete detachment of the Vero cell monolayer were studied with a phase‐contrast inverted microscope and recorded passage wise. To count dead Vero cells in this study, a protocol (0.4% trypan blue with a hemocytometer method) employed in another study [21] was followed, and the approximate percentage of dead cells was mentioned in the Results section.

#### 2.2.4. Confirmation of PPR Viral Antigen by RT‐PCR

In RT‐PCR, a fragment of the Fusion (F) and Nucleoprotein (N) genes of purified PPR viral antigen and RNA used or measured with spectrophotometry were amplified with the expected band sizes of 448 and 351 bp.

#### 2.2.5. Virus Titration and Tissue Culture Infective Dose (TCID_50_) Assay

To determine the load of PPR virus in TCF, virus titration was performed at ten‐passage intervals following an endpoint dilution assay, which quantified the amount of virus required to produce CPE in 50% of infected cells. The method of Reed and Muench [22] was used to calculate virus titers.

#### 2.2.6. Molecular Characterization

##### 2.2.6.1. RT‐PCR Confirmation

TCF at different passage levels was used for RNA extraction, and RT‐PCR was then performed, and the products were analyzed following the protocol of [15].

##### 2.2.6.2. Phylogenetic Analysis

PPRV‐TCF at the 60th passage level was sent to Friedrich Loeffler Institute (FLI), Germany, and two partial sequences of 1723 and 931 nucleotides for the N and F genes, respectively,, were received. They were named BD_PPR_08_cell culture_N and BD_PPR_08_cell culture_F. The original sequence of the isolate BD_PPR_08, including other related sequences, was downloaded from GenBank and used to compare with BD_PPR_08_cell culture. Lasergene DNA Star (Modules‐EditSeq and MegAlign) and MEGA (Version 5.10) software were used for sequence editing, alignment, and phylogenetic analysis.

#### 2.2.7. Quantitation of PPR Viral RNA by Spectrophotometry and Preparation of Purified PPR Viral Antigen by Density Gradient Ultracentrifugation

The PPR viral RNA was extracted or isolated with RNeasy Kits from TCF of different passage levels and the concentrations of PPR viral RNA were determined by measuring the absorbance at 260 nm (A260) in a spectrophotometer (Biocompare, USA) and calculated as follows: [RNA] (μg/mL) = 40 × Dilution Factor × OD260. Nucleic acids absorb light at a wavelength of 260 nm. An absorbance of 1 unit at 260 nm corresponds to 40 μg of RNA per mL (A260 = 1 = 40 μg/mL). Purified PPR viral antigen from TCF was prepared by density gradient ultracentrifugation (Thermo WX Ultra 80, USA) as described for Reo virus by Islam and Jones [23] with some modifications.

### 2.3. Statistical Analysis

Descriptive statistics were used to summarize the virological data obtained at different serial passage levels. Viral RNA concentrations are presented as mean ± standard deviation (SD) for passage levels in which measurements were available from multiple virus isolates (Passages 20 and 30). For the 60th passage, only a single measurement was available (Isolate F); therefore, only the observed value is reported, and measures of dispersion (SD or 95% confidence interval) were not calculated. Differences in viral RNA concentration between passage levels with multiple observations were evaluated using one‐way analysis of variance (ANOVA). The assumptions of normality and homogeneity of variances were assessed using the Shapiro–Wilk and Levene’s tests, respectively. Where the overall ANOVA was statistically significant, pairwise comparisons were planned using Tukey’s honestly significant difference (HSD) test. Because only a single observation was available at Passage 60, inferential comparisons involving this passage were not performed, and the result is presented descriptively. Virus titers, CPE characteristics, and RT‐PCR findings were summarized descriptively because these measurements were generated from individual virus isolates without replicated observations suitable for inferential statistical testing. All statistical tests were two‐sided, and statistical significance was defined *a priori* as *p* < 0.05. *p* values are reported together with the statistical test used and the corresponding data being compared. *p* values are presented according to standard reporting conventions (*p* < 0.001 for values < 0.001; three decimal places for values between 0.001 and 0.009; two decimal places for values ≥ 0.01). Statistical analyses were performed using IBM SPSS Statistics Version 26.0 (IBM Corp., Armonk, NY, USA).

## 3. Results

### 3.1. Initiation of CPE in Vero Cells

**FIGURE 1:**
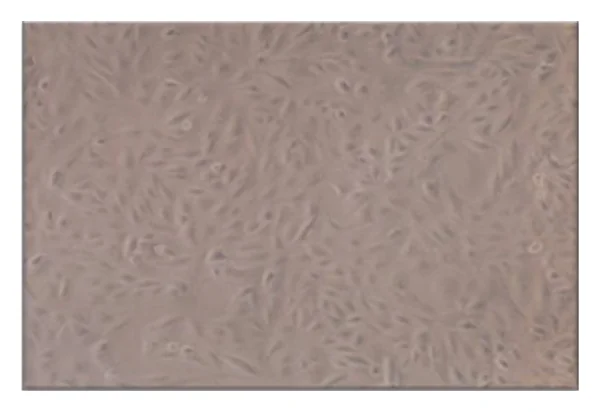
A confluent monolayer of Vero cells (control), x 82.5, phase contrast.

**FIGURE 2:**
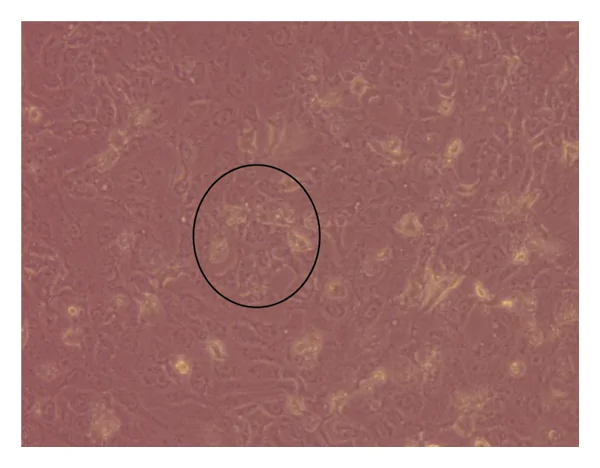
CPE (circled areas) at 24 hpi at the 60^th^ passage level: cell rounding and clustering, x 82.5, phase contrast.

**FIGURE 3:**
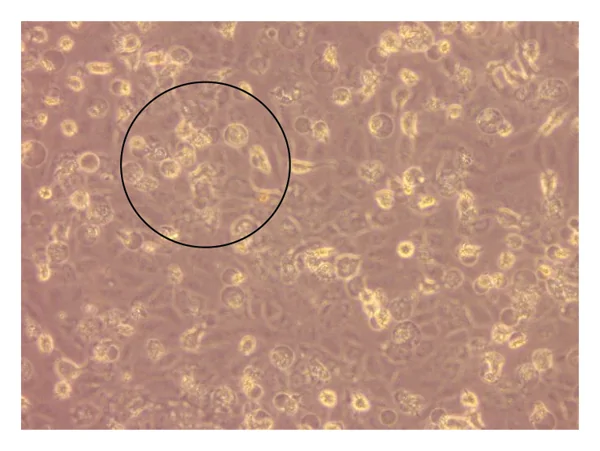
CPE (circled areas) at 36 hpi at the 60^th^ passage level: cell rounding and numerous small syncytia (2–3 cells) formation, x 82.5, phase contrast.

**FIGURE 4:**
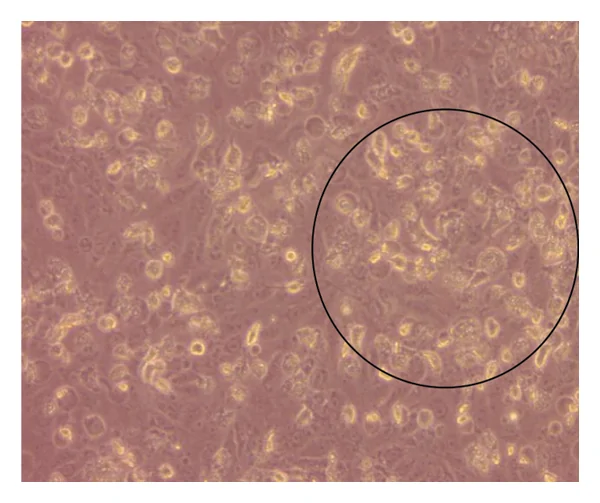
CPE (circled areas) at 48 hpi at the 60^th^ passage level: large and small syncytia, x 82.5, phase contrast.

**FIGURE 5:**
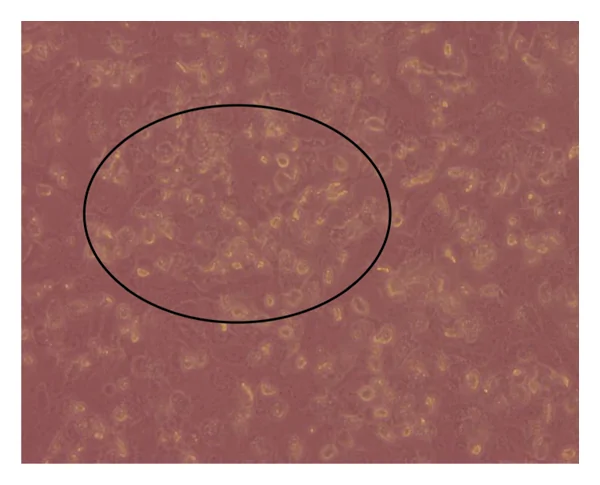
CPE (circled area) at 60 hpi at the 60^th^ passage level: almost all cells were affected and showed CPE, x 82.5, phase contrast.

**FIGURE 6:**
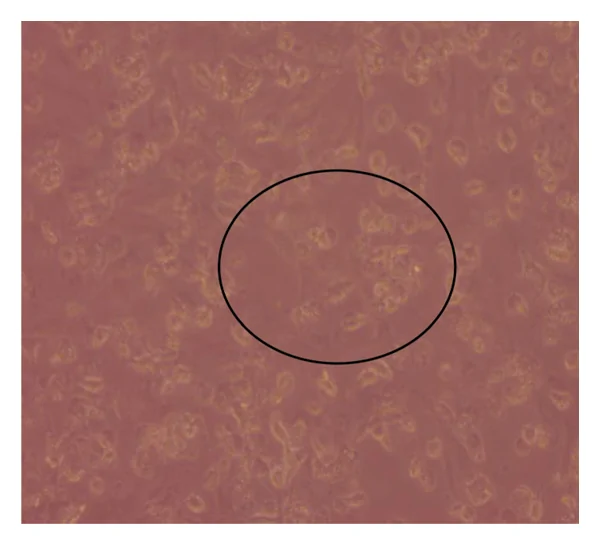
CPE (circled area) at 72 hpi at the 60^th^ passage level: almost complete detachment of the cell monolayer, x 82.5, phase contrast.

Vero cells were grown and infected with five Bangladeshi PPR virus field isolates, observed, studied, and the CPEs were documented at different passages. The CPE was not noticeable/distinguishable until 11 dpi for the first six passages in the case of all isolates. TCF and cells were harvested on Day 11 postinfection. The serial passaging was continued. The first CPE appeared at the 7th passage level for all isolates on 3 dpi (72 hpi) and was harvested on Day 8. The CPE was characterized by rounding of cells, small syncytia formation, large syncytia formation, and finally complete detachment of the cell monolayer (Figures 1, 2, 3, 4, 5, and 6).

### 3.2. Initiation of CPE Production in Lamb Kidney Primary Cells (LKC)

**FIGURE 7:**
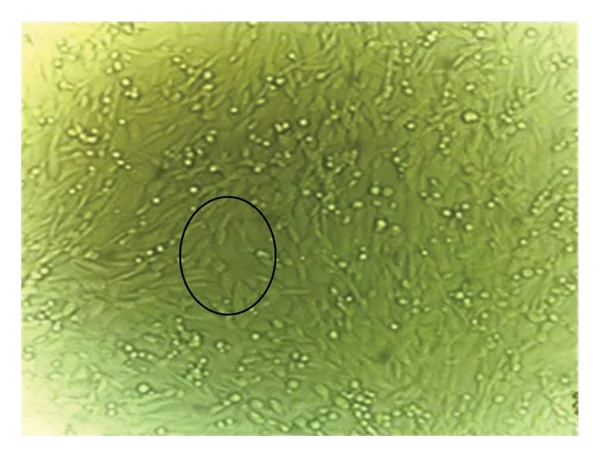
A confluent monolayer of normal lamb kidney cells (circled area), x 82.5, phase contrast.

**FIGURE 8:**
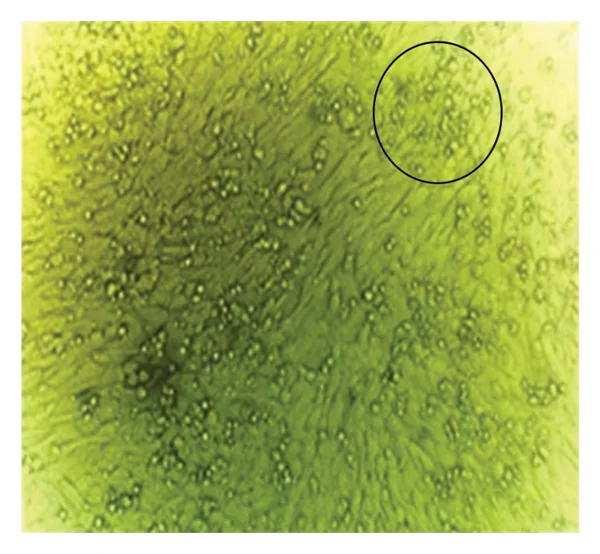
PPR virus–infected lamb kidney cells (circled area) at 24 hpi, x 82.5, phase contrast.

In lamb kidney primary cell culture, the same morphology of CPEs was observed, but rapidly, even at the first passage level, and faster in comparison to Vero cells (Figures 7 and 8).

### 3.3. The Dynamics of CPE Production (Passage‐Wise) by Different Bangladeshi Isolates of PPRV in Vero Cells

The control flasks were checked and found to be healthy. The infected flasks were harvested at 11 dpi; virus was detected in the fluid of infected flasks at the 7th passage. BD‐PPR‐08 isolate was obtained from a previous study and inoculated on Vero cells, and passaging continued with other isolates. Considering the morphology of CPE of the 10th passage, three local strains were selected for further passages until the 30th passage for BD‐PPR‐BAU‐GF‐01‐13 and BD‐PPR‐BAU‐CL‐03‐13 isolates, and the 60th passage for the BD‐PPR‐08 isolate. The presence of PPR viral RNA in TCF was confirmed by RT‐PCR and/or real time RT‐PCR at every 5‐passage interval. With the advancement of passage level, the appearance or development of CPE became faster and more progressive. The dynamics or detailed patterns of CPE production of 3 PPRV isolates are shown in Table 2.

**TABLE 2:** The dynamics of CPE production (passage‐wise) by different Bangladeshi isolates of PPRV in Vero cells.

Passage level	CPE onset	Predominant CPE characteristics	Time to complete monolayer detachment
P1–P6	No CPE	No detectable morphological changes	Not observed
P7	First detected at 5 dpi (≈72 hpi)	Initial cell rounding and separation from culture surface	Not observed
P10	24 hpi	Cell rounding (10%–20%), few floating cells, small syncytia	120 hpi
P20	24 hpi	Increased cell rounding (20%–30%), more floating cells, numerous small syncytia	96 hpi
P30	24 hpi	Extensive cell rounding (25%–35%), abundant syncytia, rapid CPE progression	84 hpi
P40	Similar to P30	Comparable CPE kinetics to P30	84 hpi
P50	≤ 12–24 hpi	Earlier onset of CPE, marked cell rounding, extensive syncytia formation	84 hpi
P60	≤ 12–24 hpi	Earliest and most severe CPE, extensive syncytia and rapid cell destruction	72 hpi

*Note:* P = passage level; hpi = hours of postinfection.

### 3.4. Time of First Detection of PPR Viral RNA in TCF by Conventional RT‐PCR

**FIGURE 9:**
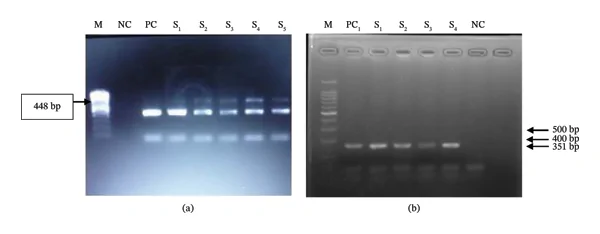
(a) Amplification of a 448 bp fragment of the F gene by RT‐PCR. S_1_ = 10^th^ passage, S_2_ = 20^th^ passage, S_3_ = 30^th^ passage, S_4_ = 40^th^ passage, and S_5_ = 50^th^ passage of PPRV‐infected TCF. Lane M: 100 bp DNA size marker, lane NC: negative control, and lane PC: positive control. (b) Amplification of a 351 bp fragment of the N gene by RT‐PCR. S_1_ = 10^th^ passage, S_2_ = 20^th^ passage, S_3_ = 30^th^ passage, and S_4_ = 40^th^ passage of PPRV‐infected TCF. Lane M: 100 bp DNA size marker, lane NC: negative control, and lane PC: positive control.

The virus was not detected in the fluid of infected and control flasks up to the 6th passage level. The first CPE appeared on five dpi at the 7^th^ passage level for all isolates. Amplification of a 448 bp fragment of the F gene (S_1_ = 10^th^ passage, S_2_ = 20^th^ passage, S_3_ = 30^th^ passage, S_4_ = 40^th^ passage, and S_5_ = 50^th^ passage) and a 351 bp fragment of the N gene (S_1_ = 10th passage, S_2_ = 20th passage, S_3_ = 30th passage, and S_4_ = 40th passage) of PPR virus infected TCF by RT‐PCR are given in Figure 9a,b.

### 3.5. Phylogenetic Analysis

**FIGURE 10:**
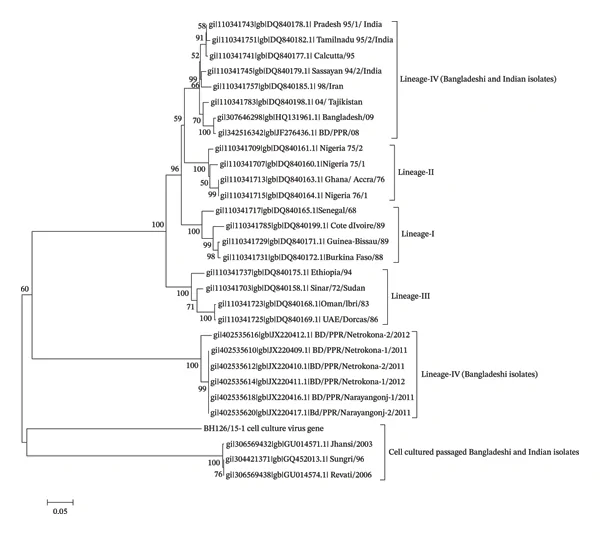
Evolutionary relationships of PPR virus isolates based on partial N gene sequences. (The evolutionary history was inferred using the neighbor‐joining method. The optimal tree with the sum of branch length = 1.88250613 is shown. The percentage of replicate trees in which the associated taxa clustered together in the bootstrap test [1000 replicates] is shown next to the branches. The tree is drawn to scale, with branch lengths in the same units as those of the evolutionary distances used to infer the phylogenetic tree. The evolutionary distances were computed using the p‐distance method and are in units of the number of base differences per site. The analysis involved 30 nucleotide sequences. Codon positions included were 1st + 2nd + 3rd+ noncoding. All positions containing gaps and missing data were eliminated. There were a total of 255 positions in the final dataset. Evolutionary analyses were conducted in MEGA 5).

The partial nucleotide sequences of the N gene of both cell culture–adapted (BH126/15‐1_cell culture) and wild (BD_PPR_08) strains of PPR virus were aligned with other Asian isolates downloaded from GenBank. A phylogenetic tree using the Neighbor‐Joining method was constructed using MEGA 5.0 software and presented in Figure 10. The serially passaged, cell culture–adapted Bangladeshi PPRV isolate BH126/15‐1_cell culture formed a well‐separated subcluster together with other serially passaged and Vero cell–adapted Indian PPR virus vaccine strains such as Sungri–96 (75 passages), Revati−2006 (50 passages), and Jhansi–2003 (50 passages).

**FIGURE 11:**
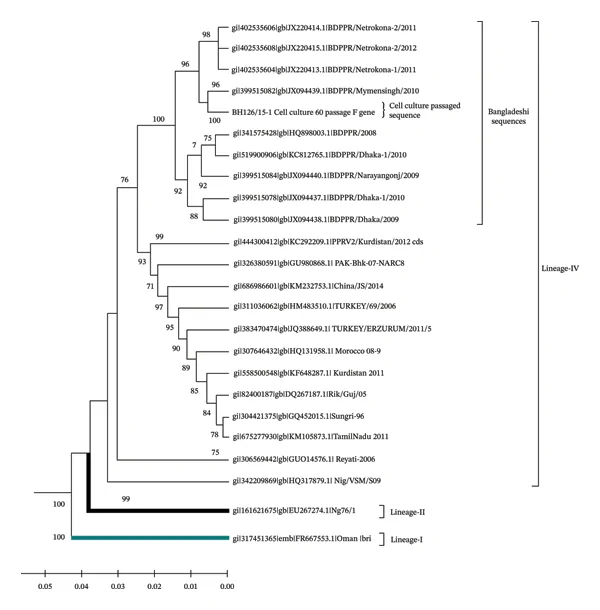
Evolutionary relationships of PPRV isolates based on partial F gene sequences. (The evolutionary history was inferred using the UPGMA method. The optimal tree with the sum of branch length = 0.30574708 is shown. The tree is drawn to scale, with branch lengths in the same units as those of the evolutionary distances used to infer the phylogenetic tree. The evolutionary distances were computed using the p‐distance method and are in units of the number of base differences per site. The analysis involved 24 nucleotide sequences. Codon positions included were 1st + 2nd + 3rd+ noncoding. All positions containing gaps and missing data were eliminated. There was a total of 249 positions in the final dataset. Evolutionary analyses were conducted in MEGA 5).

Similarly, the partial nucleotide sequences of the F gene of both cell culture–adapted (BH126/15‐1_cell culture) and wild (BD_PPR_08) strains of PPR virus were aligned with other Asian isolates downloaded from GenBank. A UPGMA phylogenetic tree was constructed using MEGA 5.0 software and presented in Figure 11. The serially passaged Bangladeshi PPR virus isolate BH126/15‐1_cell culture clustered under Lineage IV with other Bangladeshi PPRV isolates.

### 3.6. Determination of the Amount of Virus Particles in TCF at Different Passage Levels by Virus Titration

Virus titrations were performed at every 10‐passage interval for all isolates, and it was found that titers increased gradually with advancement of serial passaging with a minor variation among isolates (Table 3).

**TABLE 3:** Titers at different passage levels of different Bangladeshi isolates in Vero cells.

Isolate ID		Titers (log_10_ TCID_50_ (mL))					
	Passage no.	P‐10	P‐20	P‐30	P‐40	P‐50	P‐60
Isolate‐A		10^2.5^	10^3.2^	10^3.50^	Not done	Not done	Not done
Isolate‐B		10^2.5^	10^3.0^	10^3.41^	Not done	Not done	Not done
Isolate‐C		10^2.5^	10^3.2^	10^3.41^	Not done	Not done	Not done
Isolate‐D		10^2.5^	10^3.2^	10^3.41^	Not done	Not done	Not done
Isolate‐E		10^2.5^	10^3.0^	10^3.38^	Not done	Not done	Not done
Isolate‐F^∗^		10^2.5^	10^3.2^	10^3.55^	10^4.44^	10^4.71^	10^6.5^

*Note:* Here, P = passage level, A–F = isolate ID.

^∗^Collected from virus repository.

### 3.7. Quantitation of PPR Viral RNA at Different Passage Levels by Spectrophotometry

Mean viral RNA concentration increased from 156.67 ± 13.66 ng/µL at Passage 20 to 246.67 ± 8.16 ng/µL at Passage 30. Statistical comparisons were limited to passage levels with measurements from multiple isolates, whereas the value obtained for Passage 60 (320 ng/µL) is presented descriptively because only a single isolate was available for analysis. Details are given in Tables 4 and 5.

**TABLE 4:** Quantitation of PPR viral RNA at different passage levels of six field isolates (A–F).

Passage	Values (ng/µL)
P20	160, 150, 160, 150, 140, 180
P30	250, 240, 250, 240, 240, 260
P60	320 (only Isolate F)

*Note:* From these data, (1) P20: mean = 156.67 ng/µL. (2) P30: mean = 246.67 ng/µL. (3) P60: only one observation (320 ng/µL).

**TABLE 5:** PPR viral RNA (mean ± SD) at different passage levels of six field isolates (A–F).

Passage	Mean ± SD (ng/µL)
P20	156.67 ± 13.66
P30	246.67 ± 8.16

*Note:* Here, 95% confidence intervals (CIs) and ANOVA are not appropriate because the six values are from different virus isolates, not replicate measurements of the same experimental unit; P60 has only one observation, so SD and CI cannot be estimated.

### 3.8. Preparation of Purified PPR Viral Antigen

**FIGURE 12:**
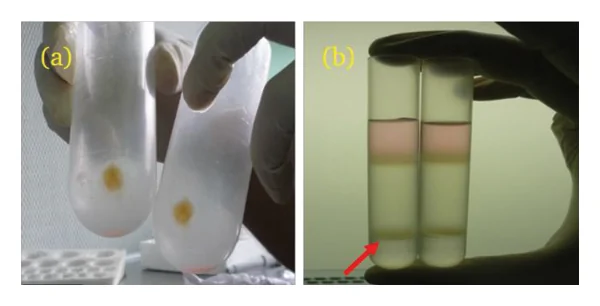
(a) Pellet of virus just after centrifugation at 50,000 g for 90 min at 4°C, and (b) a distinct layer of purified virus (red arrow) between 30% and 60% sucrose in the gradient just after centrifugation at 50,000 g for 90 min at 4°C (figure courtesy: [44]).

Purified PPR viral antigen was identified as a distinct clear band of purified virus that developed at the interface of 30% and 60% sucrose (Figure 12a,b). The PPR virus was confirmed from the prepared purified antigen by amplifying 448 bp of the F gene by RT‐PCR.

## 4. Discussion

### 4.1. CPE Patterns

This study revealed that the CPE was not noticeable until 11 dpi for the initial six passages for all PPR virus field isolates. The first CPE appeared at the 7th passage level for all isolates on 3 dpi (72 hpi) and was harvested on Day 8. All the findings are in accordance with the study conducted by [4], where the Nigeria 75/1 isolate was being passaged serially in Vero cells; the CPE was not distinguishable till 4–7 dpi for the initial 10 passages, and the TCF was harvested by 8–13 dpi. This is also in accordance with the findings of Lefevre and Diallo [24], Mohan [25] in Vero cells, and John et al. [8] in BHK cells and [10] in microcarrier culture. In Marmoset cells, CPE appeared for the first time by Day 4 at the 4th passage level with Measles virus (another antigenically related virus of the genus Morbillivirus) in a study conducted by [26]. This finding is inconsistent with the finding of this study. The variation might be due to virus species and cell line differences. In this study, CPE appeared for the first time at the 7th passage in Vero cells, though CPE was perceptible from the first passage in LKC. The findings are closest to the findings of [27], and the minor variation might be due to lineage differences and the use of an unusual host like Vero cells. First, CPE were cell rounding (10%–20% cells approximately), cells floating (2%–5%), and the appearance of small syncytia consisting of three cells under visual estimation. This is also supported by the findings of another research conducted by our research team [28]. The cytopathologic features include cell rounding, syncytia formation, intranuclear and intracytoplasmic inclusion body development, which are in unison with the statement of [11] with the Sungri strain of PPR virus on 96 hpi. The findings also support the findings of related previous studies of [29]; Lefevre and Diallo [24]; Mohan [25] in Vero cells; John et al. [8] in BHK‐21 cells; and Sreenivasa et al. [9] in Marmoset B95a cells. In this study, complete detachment of the Vero cell monolayer occurred with all Bangladeshi field isolates of PPR virus on 120 hpi at the 10th passage, 96 hpi at the 20th passage, 84 hpi at the 30th passage, and 72 hpi at the 60th passage level, which almost supports the findings of the study of [11] who described that complete dissociation of the Vero cell monolayer occurred with the Arasur strain of PPR vaccine virus by 120 hpi. The differences might be due to the passage level or adaptation level of PPR virus in Vero cells, though Hegde did not mention the passage level. Those CPE are also in agreement with other Paramyxoviruses such as canine distemper virus in Vero cells [30], in Marmoset B95 cells [31], and chick embryo fibroblast monolayer tissue culture [32], Rinderpest virus in Vero cells [33], and Measles virus in Vero cells. Percentages of infected Vero cells at different hpi of different passage levels were identified by visual estimation under phase contrast inverted microscope—though this is a limitation of this study but the findings are very resembling to other studies stated above using an unusual host like Vero cells, though in LKC, CPE initiations are faster and rapid. A titer of 6.3 log_10_ TCID_50_/mL [34] is comparable to the titer of 6.5 log_10_ TCID_50_/mL observed in the present study, whereas the value reported by Asim et al. [35] (5.2 log_10_ TCID_50_/mL) is notably lower. Titer (5.5 log 10 TCID50/mL) in the present study was found close to a titer of 5.83 log 10 TCID50/mL for the Sungri strain of PPR virus in Vero cells, where the average titer of the PPR vaccine was 10^6.29^/mL and 10^5.58^/mL in chilled and freeze‐dried vaccine, respectively, by Hegde et al. [11, 36]. The titer was found to be 5.5 log 10 TCID50/mL in the study conducted by Abbas et al. [5] with PPR virus (Nigeria 75/1 strain), which supports the findings of this study.

### 4.2. RNA Quantification

The concentration of PPR viral RNA at different passage levels was quantified in this study, which found 156.66 and 246.66 ng/µL at the 20th and 30th passage levels, respectively, as the average for the five isolates (A–E) and 320 ng/µL at the 60th passage for isolate‐F by spectrophotometry, which supports methodologically the study conducted by [37] in a study that compared the concentration of viral, prokaryotic, and RNA among different methods and Courcelle [38], who measured measles and rinderpest viruses by spectrophotometry, finding that viral RNA concentrations varied significantly depending on the stage of infection and the tissue type sampled. These variations underscore the importance of context when interpreting viral load, as different tissues can harbor different viral concentrations, affecting the overall assessment of the disease state. Research by Manjunath [39] emphasized the need for accurate viral quantification to inform vaccine development and disease control strategies, supporting this study and another study by the same research group [29]. RNA concentration and viral titer across passage levels correspond and indicate gradual adaptation of the PPR virus in Vero cells. This may be due to mutation of the F gene, resulting in loss of viral pathogenicity. Gradual increment of both concentration and viral titer is important for vaccine development strategy, which supports another study of this research team [40]. The PPR viral RNA concentration in this study is found to be less than that of [41], who reported that another Morbillivirus, measles virus, can reach concentrations of approximately 500 ng/µL in peripheral blood mononuclear cells (PBMCs) during peak viral replication, indicating more vigorous replication in certain cell types during acute infection. This variation may be due to acute infection, but our study was based on the adaptation of PPR virus in an unusual host, such as Vero cell culture. Research has demonstrated that the measles virus can reach concentrations of approximately 500 ng/µL in PBMCs during peak viral replication [41]. This concentration is notably higher than the PPRV concentrations reported, indicating more vigorous replication in certain cell types during acute infection. Studies have reported FMDV RNA concentrations ranging from 100 ng/µL to over 1000 ng/µL in vesicular fluids and tissues during acute infections [42]. This suggests that FMDV can achieve significantly higher concentrations than those observed for PPR virus, particularly in tissues directly involved in viral replication. Studies have shown that rabies viral RNA concentrations can reach up to 300 ng/µL in brain tissues during the terminal stages of the infection [43]. While this concentration is comparable to the highest observed PPR virus concentration (320 ng/µL for isolate F), it is important to note that rabies primarily affects the central nervous system, which may influence the distribution and quantification of viral RNA.

### 4.3. Antigen Purification

Purified PPR viral antigen was prepared in this study after measuring viral RNA concentration in TCF, which is in accordance with the strategy and findings of a study conducted by [44] and was used in different molecular techniques, such as RT‐PCR, as a positive control. Density gradient ultracentrifugation was used in this study to purify PPR viral antigen, which is in accordance with the study of [45, 46], which ensures high antigenicity for diagnostic and vaccine applications. Recent advancements include the use of recombinant goat pox virus vectors expressing PPR antigens, which improve the efficiency of antigen delivery and immune response [47, 48]. Purified Measles virus antigens were prepared by cultivating the virus in human diploid cells or Vero cells [49] and FMD virus was cultured in BHK‐21 cells or primary calf thyroid cells and purified FMD virus antigen. Both studies support the protocol, purification, and application of PPR antigen produced in this study.

### 4.4. Implications

The findings surrounding PPR viral RNA concentrations present a foundation for further research into the dynamics of viral shedding and transmission. Understanding these dynamics can help in developing better control measures for PPR and related viruses. Additionally, integrating spectrophotometric data with other quantification methods, such as quantitative PCR (qPCR), could provide a more comprehensive view of viral loads and their implications for disease management. The hypothesis of this study also supports the study of Parida [50].

## 5. Conclusion

It is concluded that recent Bangladeshi PPRV field isolates required adaptation to Vero cells, with consistent CPE development and RT‐PCR detection first occurring at the 7th passage. Serial passaging significantly increased viral RNA concentration and infectious virus titer, indicating enhanced viral adaptation. TCF from higher passages, particularly the 60th passage, yielded the highest‐quality viral antigen and represents the most suitable source for antigen purification, supporting future diagnostic assay development and PPRV vaccine research.

## Author Contributions

M. S. I. Siddiqui: conceptualization, methodology, investigation, data analysis validation, writing of the original manuscript, review and editing of the manuscript. Jahan Ara Begum: conceptualization, methodology, investigation, validation, review and editing of the manuscript. M. R. Islam and E. H. Chowdhury: conceptualization, methodology, project administration, supervision, resources, funding acquisition, formal analysis, validation review and editing of the manuscript.

## Funding

The Bangladesh Agricultural Research Council (BARC), Ministry of Agriculture, Government of the People’s Republic of Bangladesh (grant numbers SPGR‐360, NATPPhase‐1, and BARC), supported this work.

## Disclosure

All authors have read and approved the final version of the manuscript. The first author of the manuscript affirms that this manuscript is an honest, accurate, and transparent account of the study being reported; that no important aspects of the study have been omitted; and that any discrepancies from the study as planned have been explained. The corresponding author had full access to all of the data in this study and takes complete responsibility for the integrity of the data and the accuracy of the data analysis.

## Ethics Statement

The authors have nothing to report.

## Consent

The authors have nothing to report.

## Conflicts of Interest

The authors declare no conflicts of interest.

## Data Availability

The datasets generated and/or analyzed during the current study are available from the corresponding author on reasonable request.
